# The role of chidamide in the treatment of B-cell non-Hodgkin lymphoma: An updated systematic review

**DOI:** 10.17305/bb.2023.8791

**Published:** 2023-10-01

**Authors:** Hastono Ridwansyah, Indra Wijaya, Muhammad Hasan Bashari, Achmad Hussein Sundawa Kartamihardja, Bethy Suryawathy Hernowo

**Affiliations:** 1 Doctoral Study Program, Faculty of Medicine, Universitas Padjadjaran, Bandung, Indonesia; 2 Department of Biomedicine, Faculty of Medicine, President University, Bekasi, Indonesia; 3 Division of Hematology and Oncology, Department of Internal Medicine, Faculty of Medicine, Universitas Padjadjaran, Hasan Sadikin General Hospital, Bandung, Indonesia; 4 Department of Biomedical Sciences, Faculty of Medicine, Universitas Padjadjaran, Bandung, Indonesia; 5 Department of Nuclear Medicine and Molecular Theranostic, Faculty of Medicine, Universitas Padjadjaran, Bandung, Indonesia; 6 Department of Anatomical Pathology, Faculty of Medicine, Universitas Padjadjaran, Bandung, Indonesia

**Keywords:** Chidamide, tucidinostat, histone deacetylase (HDAC), histone deacetylase inhibitors, non-Hodgkin lymphoma (NHL)

## Abstract

B-cell non-Hodgkin lymphoma (B-NHL) is a lymphoid malignancy derived from B-cells that remains difficult to treat. Moreover, relapses and refractory cases are common. Abnormalities in epigenetic mechanisms, such as imbalanced histone acetylation affecting certain genes, contribute to relapses and refractory cases. Chidamide (tucidinostat) is a novel histone deacetylase inhibitor that can reverse this epigenetic imbalance and has been approved for the treatment of T-cell malignancies. However, the use of chidamide for B-NHL remains limited, and the lack of relevant literature exacerbates this limitation. We conducted this review to summarize the anticancer activity of chidamide against B-NHL and its clinical applications to overcome drug resistance. This systematic review was conducted according to the PRISMA 2020 guidelines, using some keyword combinations from MEDLINE and EBSCO. The inclusion and exclusion criteria were also defined. Of the 131 records retrieved from databases, 16 were included in the review. Nine articles revealed that chidamide limited tumor progression by modifying the tumor microenvironment, stopping the cell cycle, inducing apoptosis and autophagy, and enhancing complement-dependent and antibody-dependent cell-mediated cytotoxicities. According to seven other studies, administering chidamide in combination with another existing therapeutic regimen may benefit not only patients with relapsed/refractory B-NHL but also those with newly diagnosed B-NHL. Chidamide plays many important roles in limiting B-NHL progression through epigenetic modifications. Thus, combining chidamide with other anticancer drugs may be more beneficial for patients with newly diagnosed and relapsed/refractory B-NHL.

## Introduction

Non-Hodgkin lymphoma (NHL) is a hematological malignancy of the lymphatic system that shows favorable response to the existing treatments, but some cases remain difficult to treat. Some patients with B-cell NHL (B-NHL) relapse and develop resistance to standard immunochemotherapies [1–3]. Standard drug resistance forces doctors to intensify recommended therapy, putting patients at risk of side effects, and necessitating a breakthrough to solve the problems [4].

Approximately 90% of NHL is derived from B-cells [5]. A fundamental understanding of B-NHL pathogenesis will lead to the development of a new drug to treat the disease. Some theories about the triggers of relapsed and refractory B-NHL to some therapeutic modalities have emerged [3, 5]. One of the many processes underlying the disease is thought to be an epigenetic mechanism [3, 6, 7]. Some histone-modifying genes can drive tumor progression in diffuse large B-cell lymphoma (DLBCL). Inactivation of cyclic adenosine monophosphate response element-binding protein binding protein (*CREBBP),* which encodes a histone acetyltransferase, results in a lack of histone acetylation and plays a significant role in lymphomagenesis [8, 9]. For instance, mutations or genomic loss in *CREBBP* and *EP300* in follicular lymphoma (FL) are associated with disease relapse and poor prognosis [10, 11].

Histone deacetylase (HDAC) is an enzyme that deacetylates histones, altering chromatin conformation and preventing transcription. It is comprised of four classes: HDAC Class I (HDAC1, 2, 3, and 8); IIa (HDAC4, 5, 7, and 9); IIb (HDAC6 and 10); III (SIRT1–7); and IV (HDAC11). These enzymes are reported to mediate antitumor gene silencing, leading to unfavorable outcomes in patients with B-NHL. Some studies have revealed that HDAC2, 3, and 5 were associated with poor clinical outcomes in DLBCL subtypes [10, 11]. However, the patient’s poor clinical outcomes have yet to be comprehensively explained.

Chidamide is a novel HDAC inhibitor (HDACI) that modifies the epigenetic mechanism, inhibits tumor growth by inhibiting HDAC1, 2, 3, and 10 enzymes, and rescues histone acetylation deficits [12–14]. It was first introduced and approved by the China Food and Drug Administration for treating T-cell lymphoma because of its efficacy and safety [15]. In addition to peripheral T-cell lymphoma, chidamide was approved in postmenopausal patients with hormone receptor-positive HER2-negative advanced breast cancer by the National Medical Product Administration of China. Moreover, chidamide was approved for treating relapsed or refractory adult T-cell leukemia–lymphoma and relapsed or refractory peripheral T-cell lymphoma by the Ministry of Health, Labour, and Welfare of Japan (Hiyasta^®^ ). Furthermore, chidamide has been reported to enhance the efficacy of existing standard treatments for certain types of hematological malignancies [16–18].

There have been no systematic reviews on the role of chidamide in the treatment of B-NHL as a new drug, from its mo lecular mechanism of action to its clinical application. This systematic review updates and summarizes the role of chidamide as an anticancer agent in patients with new and relapsed/refractory (R/R) B-NHL using specific mechanisms. In addition, recommendations and future challenges regarding chidamide utilization are presented.

## Materials and methods

### Search and selection strategy

A systematic literature search was conducted for all relevant studies according to the updated Preferred Reporting Items for Systematic Reviews Meta-Analysis (PRISMA) guidelines [19]. To identify newly published articles, the following keywords were entered in MEDLINE and EBSCO electronic databases: “Chidamide,” AND “non-Hodgkin Lymphoma” AND “B-cells” in August 2022 and again in October 2022. Additionally, different keywords were used to search the literature in MEDLINE, as follows: [(“chidamide” AND “diffuse large B-cell Lymphoma”) OR (“chidamide” AND “follicular lymphoma”) OR (“chidamide” AND “Burkitt lymphoma”) OR (“chidamide” AND “Mantle Cell Lymphoma”) OR (“chidamide” AND “MALT lymphoma”) OR (“chidamide” AND “marginal zone B-cell lymphoma”)]. Because chidamide is also known as tucidinostat, HBI-8000, or Hiyasta^®^, we expanded the literature search by including those names in the MEDLINE, EBSCO, and Google Scholar search to obtain the relevant literature. These keywords were used to search for articles published between 2012 and 2022. The inclusion criteria in this analysis were a research article on B-NHL written in English, as determined by the title and abstract of each article, whereas the exclusion criteria were case reports and studies that did not use chidamide. The synthesis was based on preclinical and clinical study articles in which the quality of the included studies had been assessed.

### Data extraction

Data were extracted using a standardized form that was approved by all authors. Data extracted from preclinical studies included: (1) authors and year of publication, (2) types of in vitro models used in the experiment, (3) mechanism of action of chidamide for treating B-NHL, and (4) treatment, whereas data extracted from clinical studies included: (1) number of participants, (2) disease diagnosis, (3) median age of participants, (4) interventions, and (5) participant outcomes, including efficacy and safety of each regimen.

### Risk of bias assessment

In this review, the risk of bias in clinical research articles was assessed using the risk of bias in non-randomized studies of interventions (ROBINS-I) assessment tool [20–23]. All authors were involved in assessing the risk of bias in all included articles. The risk of bias for in vitro studies was not assessed because none of the quality assessment tools was suitable for assessing all critical aspects of systematic reviews of in vitro studies [22].

## Results

**Figure 1.**
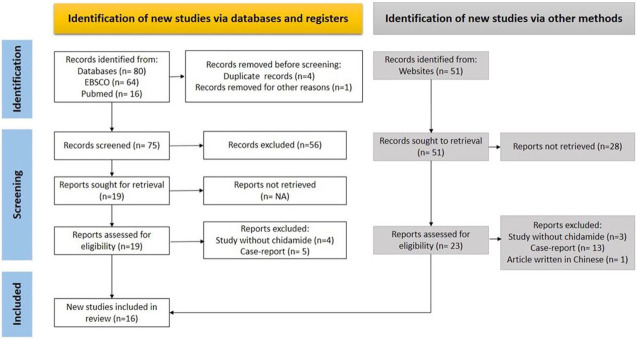
**A systematic literature search was conducted using a PRISMA flow diagram.** Of the 131 articles retrieved, 16 were included in the review. PRISMA: Preferred Reporting Items for Systematic Reviews Meta-Analysis.

The results of the systematic literature search are depicted in Figure 1. Eighty articles were retrieved from two database sources, with one excluded due to the lack of an abstract section and 56 excluded since they were not research articles on B-NHL. Nine out of the 19 articles retrived were excluded because the studies did not use chidamide or were case reports. Six additional articles identified using other methods were included. As a result, 16 articles were finally included in this review.

The majority of studies on the mechanism of action of chidamide have been conducted using various R/R B-NHL cell lines and tumor xenograft models (Table 1). The results describing the mechanism of action of chidamide as an anticancer agent for B-NHL are detailed in the next section.

**Table 1:** Characteristic of the preclinical studies on the main mechanism of action of chidamide as an antitumor agent for B-cell non-Hodgkin lymphoma

**References**	**Preclinical models**	**Principal mechanism of action**	**Treatment**
Zhang et al. 2021	ABC-DLBCL and GCB-DLBCL cell lines	Inhibiting HDAC2/STAT3/BCL-2 signaling pathway	Chidamide
Zhong et al. 2021	Transformed FL cell lines and a tumor xenograft model	Inducing G1 arrest and apoptosis by suppressing the PI3K/PDK1/AKT pathway	Chidamide
Bissonnette et al. 2021	A20 B-cell lymphoma mice xenograft model	Enhancing the functions of dendritic and antigen-presenting cells, and modulation of MHC class I and II	Chidamide and immune checkpoint inhibitors
Xue et al. 2021	R/R Burkitt lymphoma and DLBCL cell lines and a tumor xenograft model	Increasing cleaved caspase 3 and 8 and PARP Upregulating *FOXO1* and *BTG1* gene expression	Chidamide alone and in combination with cisplatin, etoposide, or gemcitabine
Guan et al. 2020	DLBCL cell lines, a mouse xenograft model, and patient	Upregulating CD20 mRNA and protein expression by inhibiting HDAC3	Chidamide and rituximab
Yang et al. 2021	DLBCL, MCL, BL, B-ALL, and B-lymphoblastic lymphoma cell lines and a xenograft model	Inhibiting HDAC and increasing CD22 expression on the cell surface	Chidamide and CAR-T cell
Luo et al. 2022	DLBCL cell lines and a mouse xenograft model	Downregulating MYC, *BCL2*, and *TP53* expressions	Chidamide and venetoclax
Fang et al. 2018	Double BCL2/MYC expression of DLBCL patient-derived xenograft models	Regulating a set of genes involved in regulation of BCL2 and MYC	Chidamide and doxorubicin
Wang et al. 2021	DLBCL cell lines and a mouse xenograft model	Reducing ORC1 of the DNA replication process	Chidamide and EZH2 inhibitor SHR2554

ABC: Activated B-cell; DLBCL: Diffuse large B-cell lymphoma; GCB: Germinal center B-cell; HDAC2: Histone deacetylase 2; STAT3: Signal transducer and activator of transcription 3; BCL-2: B-cell lymphoma 2; FL: Follicular lymphoma; PI3K/PDK1/AKT: Phosphatidylinositol-3 kinase/Phosphoinositide-dependent kinase-1/Protein kinase B; MHC: Major histocompatibility complex; R/R: Relapsed/refractory; PARP: Poly (ADP-ribose) polymerase; BTG-1: B-cell translocation gene 1; MCL: Myeloid cell leukemia 1; B-ALL: B-cell acute lymphoblastic leukemia; BL: Burkitt lymphoma; CAR-T: Chimeric antigen receptor T-cell; ORC1: Origin recognition complex subunit 1; EZH2: Enhancer of zeste homolog 2.

The main mechanisms of action of chidamide in the B-NHL are summarized in Table 1, whereas data on efficacy, safety, and utilization of chidamide over the last decade are presented in Table 2. Six clinical studies and one observational retrospective study were conducted with a total of 203 participants who received chidamide in the last decade. Chidamide was given either alone or in combination with immuno- and chemotherapies. Patients with B-NHL in many different trials received various doses of chidamide, with 10 mg given in one study [23], 20 mg in two studies [24, 25], 30 mg in four studies, and 40 mg in one study [26–29].

**Table 2:** Characteristics of the clinical and observational studies on the clinical application of chidamide in treating patients with B-cell non-Hodgkin lymphoma

**References**	**Participants and median age**	**Intervention**	**Outcomes**
Chen et al. 2021	10 and 3 elderly patients with R/R DLBCL and FL; DLBCL: 72.5 and FL: 64 years old	Chidamide and rituximab	Efficacy: DLBCL: CR 30%, PR 10%, PD 60%; FL: CR 33.3%, and PR 66.7% Safety: About 15% of patients had grades III/IV AEs
Zhang et al. 2020	49 elderly patients with newly diagnosed DLBCL; 67 years old	Chidamide and R-CHOP	Efficacy: CR 86%; ORR 94%; 2-year PFS 68%; and 2-year OS 83%. Safety: Grade IV neutropenia 53% and grade IV thrombocytopenia 2%
Wang et al. 2021	34 patients with R/R DLBCL with previous treatments; 66 years old	Chidamide and PEL	Efficacy: CR 35.3%, PR 14.7%, SD 8.8%, PD 42.1%, and ORR 50%. Safety: Grade III/IV neutropenia 11.2% and grade III/IV anemia 4.19%
Ji et al. 2021	60 patients with high-risk or R/R B-NHL; 35 years old	Chidamide and CGB after transplantation with autologous stem cells	Efficacy: CR 100%; OS (35.4 months) 94.5%; and 4-year PFS 88.2% Safety: Grade III: mucositis 19%, dermatitis 20%, and diarrhea 11.4%; Grade IV: sepsis 1.9%
Qu et al. 2019	13 patients with R/R DLBCL; 56 years old	Chidamide and decitabine plus rituximab, gemcitabine, and oxaliplatine	Efficacy: ORR 100% and CR 23.1% Safety: Grade III/IV: thrombocytopenia 76.9%, anemia 46.2%, neutropenia 92.3%, and diarrhea 46.2%
Sun et al. 2021	20 patients with R/R DLBCL; 51 years old	Chidamide	Efficacy: ORR 25% and CR 15% Safety: Not reported
Yoshimitsu et al. 2022	14 patients with non-Hodgkin lymphoma	Chidamide	Efficacy: In 30 mg BIW cohort: ORR 16.7% and disease control 66.7%; In 40 mg BIW cohort ORR 71.4% and disease control 100% Safety: Grade III/IV TEAE 78.6%; MTD: 40 mg BIW of chidamide

R/R: Relapsed/refractory; DLBCL: Diffuse large B-cell lymphoma; FL: Follicular lymphoma; CR: Complete response; PR: Partial response; PD: Persistent disease; SD: Stable disease; ORR: Overall response rate; R-CHOP: Rituximab, cyclophosphamide, hydroxydaunorubicin, Oncovin, prednisone; PEL: Prednisone, etoposide, lenalidomide; B-NHL: B-cell non-Hodgkin lymphoma; CGB: Cladribine, gemcitabine, busulfan; PFS: Progression-free survival; OS: Overall survival; AEs: Adverse events; TEAE: Treatment-emergent adverse event; MTD: Maximum tolerated dose.

The clinical outcomes of B-NHL patients evaluated after administration of chidamide included the response rate and overall and progression-free survival, as reported in Table 2. In fact, the overall response rate after administration of chidamide in patients receiving drug combinations reached 40%–100% compared with the response rate in patients receiving chidamide alone (25%). The majority of chidamide recipients were over 50 and had relapsed or refractory B-NHL (DLBCL and FL subtypes) with various drug combinations. Chidamide was given to 49 patients when they were first diagnosed with B-NHL.

Many mild to serious adverse events (AEs) have been reported following chidamide-containing regimens administration. Aside from anemia and thrombocytopenia, neutropenia was the most frequent hematological AE, whereas nonhematological AEs included gastrointestinal tract symptoms (nausea, vomiting, and diarrhea), infection or sepsis, and fatigue. The majority of AEs in patients who received chidamide-containing regimens were mild.

**Figure 2.**
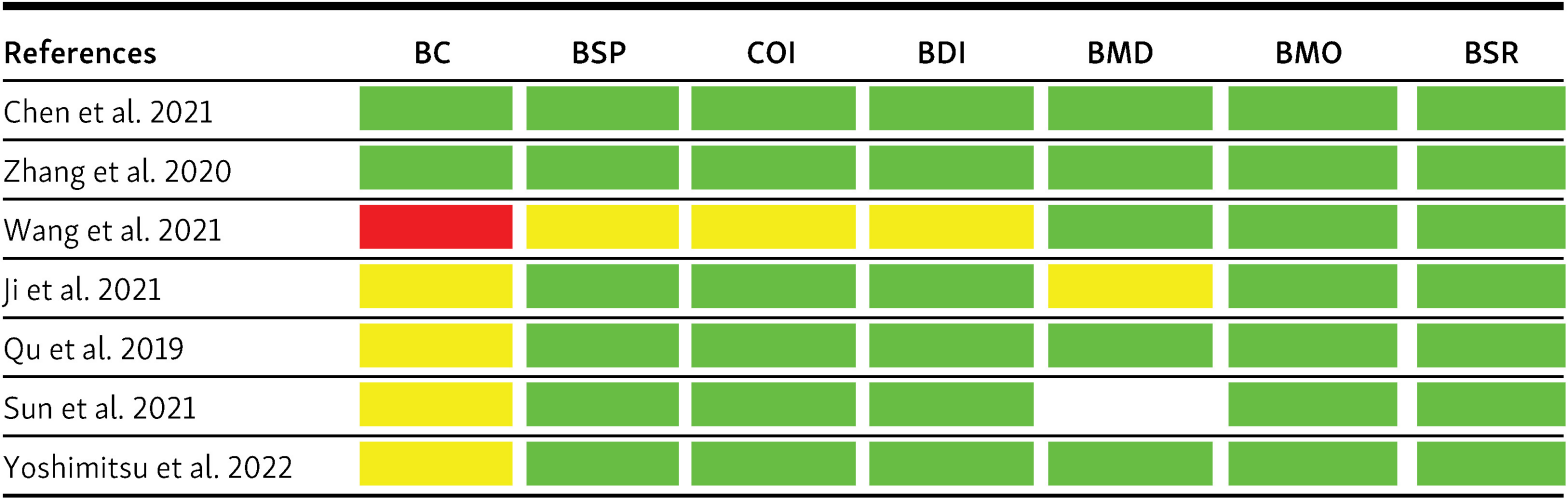
**Risk of bias assessment summary using ROBINS-I.** BC: Bias due to confounding; BSP: Bias in the selection of participants; COI: Bias in the classification of interventions; BDI: Bias due to deviations from the intended intervention; BMD: Bias due to missing data; BMO: Bias in the measurement of outcomes; BSR: Bias in the selection of the reported result; ROBINS-I: Risk of bias in non-randomized studies of interventions. *Green*: low risk of bias; *yellow*: moderate risk of bias; *red*: serious risk of bias; and *white*: no information.

Among six articles, five clinical trial articles and one retrospective cohort article were assessed for risk of bias using ROBIN-I tool (Figure 2). After being assessed for overall domains, the majority of clinical trials were classified as having a low risk of bias [25, 26, 28–30]. This finding indicates that the studies were comparable to a well-performed randomized trial. Each article had a moderate risk of bias in the “bias due to confounding” domain. However, the studies still provided sound evidence for a nonrandomized study but cannot be comparable to a well-performed randomized trial [20]. There was no critical risk of bias in any of the articles. Therefore, all articles were included in further discussion and synthesis.

## Discussion

The majority of NHL cases are B-cell subtypes, and DLBCL is the most common subtype of mature B-NHL, accounting for 30%–40% of all new diagnoses [31, 32]. Several studies have shown that resistance to existing standard B-NHL regimens is common [33]. Resistance to rituximab–cyclophosphamide–hydroxydaunorubicin–Oncovin–prednisone (R-CHOP) occurred in 30% of patients with DLBCL, thereby increasing mortality [34]. This incidence was inextricably linked to epigenetic mechanisms, with approximately 25% of patients with DLBCL having loss-of-function mutations in histone acetyltransferase-encoding genes, such as *CREBBP* and *EP300*. Patients with FL who relapsed frequently had a high proportion of mutations in these genes as well [35]. The impact of this mutation is a negative imbalance in which HDAC predominates in deacetylating histone, altering the chromatin topology to become highly condensed and suppressing transcription of some genes, thereby allowing tumor progression [36].

### Mechanism of action of chidamide

Some pathways involving many proteins can contribute to lymphomagenesis and drug resistance in NHL. Some of these processes appear to be based on epigenetic mechanisms that control intracellular conditions to inhibit apoptosis, stop the cell cycle, increase cell survival and proliferation, and interfere with the optimal functioning of existing therapeutic modalities. The underlying mechanisms by which chidamide limits B-cell lymphoma progression are as follows.

#### Cell cycle arrest

**Figure 3.**
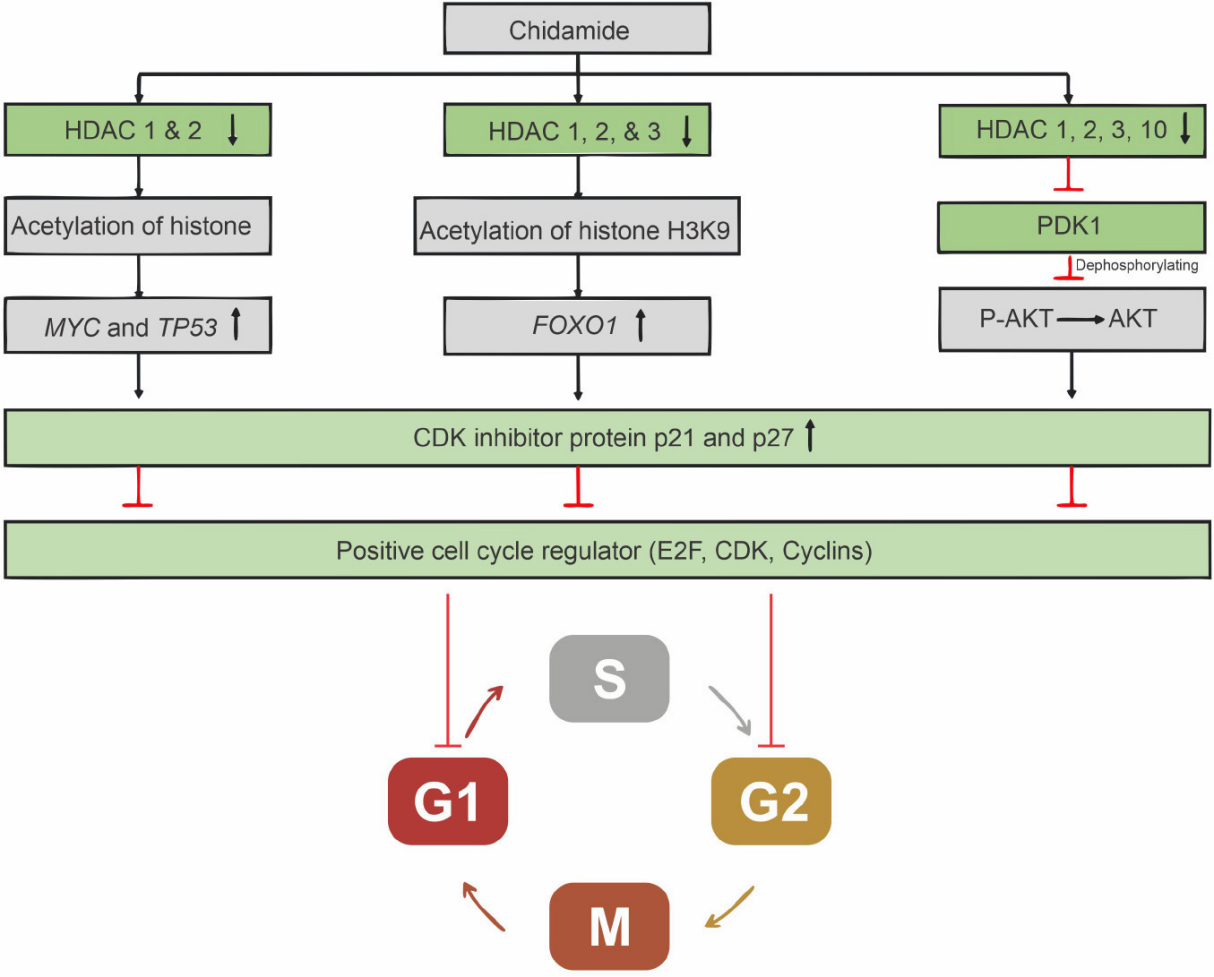
**Proposed mechanisms of action of chidamide in inducing cell cycle arrest in B-cell non-Hodgkin lymphoma.** HDAC: Histone deacetylase; CKI: Cyclin-dependent kinase.

Chidamide can stop the cell cycle of B-NHL via several reported mechanisms (Figure 3). The PI3K/AKT pathway is continuously activated during lymphoma progression due to a high rate of AKT phosphorylation at Serine 473 (Ser473) and Thr308 by a protein kinase known as PDK1 [37–39]. Chidamide can dephosphorylate AKT on Ser473 and Thr308 by inhibiting PDK1 expression, and inactivation of the PI3K/AKT signaling pathway promotes cell cycle arrest in DLBCL [40]. Chidamide, at a concentration of 5 µM, was reported to inhibit HDAC1, 2, 3, and 10 as well as promote histone H3 acetylation, which is consistent with another study conducted to transform FL. During the acetylation process, PDK1 and AKT expression levels decreased significantly. The low expression of PDK1, which is probably associated with the expression levels of HDAC1, 2, 3, and 10, has an important role in increasing the cyclin-dependent kinase (CDK) inhibitor (CKI) p27, which then causes tumor cells to stop their cycle in the G0/G1 phase [40]. Several studies have also reported that increased HDAC1 and 2 expressions were associated with low p21 and p57, whereas high HDAC3 expression was associated with low p27 in activated B-cell-like (ABC) DLBCL. Chidamide can increase those CDK inhibitors, thereby ceasing the cell cycle [41, 42].

Another study found that chidamide (3 µM) could induce cell cycle arrest at the G0/G1 phase in R/R DLBCL and Burkitt lymphoma by altering gene expression profiling. Chidamide promoted gene expression alteration by amplifying histone H3K9 acetylation on *FOXO1* promoter regions. *FOXO1* gene expression appears to increase p21/p27 while decreasing cyclin A2, CDK2, and cyclin B1 at both the mRNA and protein levels. Chidamide increases p21 and p27 expressions while decreasing the cell cycle-positive regulators E2F, cyclin, and CDK at both the transcriptional and translational levels, resulting in cell cycle arrest not only in the G0/G1 phase but also in the G2/M phase [43].

Chidamide alone could only inhibit HDAC1 and 2 in the SUDHL-4 DLBCL cell line. However, it could inhibit more HDAC enzymes, such as HDAC7 and 10, when combined with venetoclax, a highly selective small-molecule BH3 mimetic with a greater affinity for BCL-2. Inhibiting these enzymes caused an increase in MYC and TP53 mRNA and protein expression. Both were eventually able to activate the CDK inhibitors, allowing B-cell lymphoma to stop the cell cycle in the G0/G1 phase [44].

#### Inducing cell death through apoptosis and autophagy

**Figure 4.**
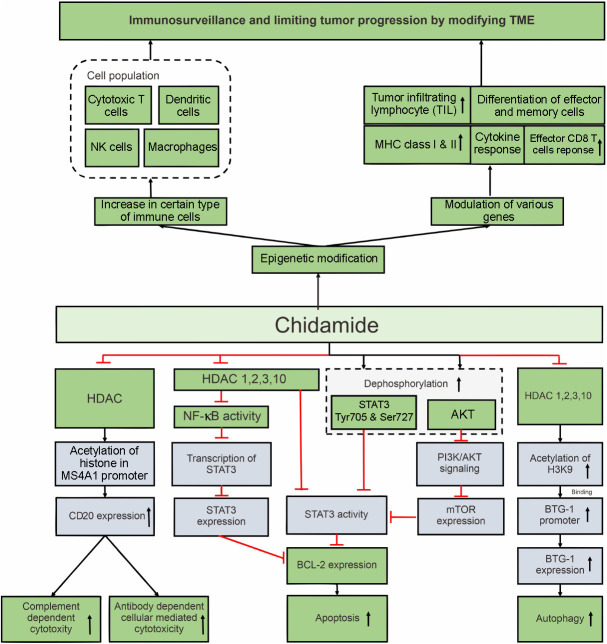
**Mechanisms by which chidamide promotes cell death and modulates TME in B-cell non-Hodgkin lymphoma through various mechanisms.** TME: Tumor microenvironment; HDAC: Histone deacetylase; MHC: Major histocompatibility complex; NF-κB: Nuclear factor kappa B; STAT3: Signal transducer and activator of transcription 3; BTG-1: B-cell translocation gene-1; NK: Natural killer.

Some studies reported that some standard treatment modalities failed to induce tumor cell death in B-NHL, resulting in refractory cases. The mechanisms underlying tumor cell failure in programming cell death have been elucidated, suggesting a potential molecular target for chidamide treatment (Figure 4).

DLBCL, an aggressive B-NHL subtype, has a high expression of antiapoptotic BCL-2, which can prevent apoptosis in tumor cells. Targeting BCL-2 is expected to overcome therapeutic resistance in B-NHL [45]. The high expression of this protein is associated with the increased expression and phosphorylation of signal transducer and activator of transcription 3 (STAT3) in lymphoma B-cells, which in turn promotes DLBCL progression [46]. The STAT3 protein is a novel molecular mediator of chemoradiotherapy resistance in some cancers [47]. The ABC-DLBCL subtype is more resistant to R-CHOP when it expresses more STAT3 than germinal center B-cell (GCB)-DLBCL [48–50]. Chidamide can reduce and dephosphorylate STAT3 in DLBCL cells in a dose-dependent manner by inhibiting HDAC1, 2, 3, and 10. It was supposed to reduce STAT3 mRNA and protein expression levels in the B-NHL cell line in a dose-dependent manner during the first 48 h of treatment [51].

Another proposed mechanism for high STAT3 expression in some kinds of malignancies is nuclear factor kappa B (NF-κB) activity [52], which can physically interact with *STAT3* promoters to modulate its expression [53–56]. Chidamide, by inhibiting HDACs and decreasing NF-κB transcriptional activity, can suppress STAT3 and slow tumor progression [57, 58]. The downstream effector of STAT3, BCL-2, which acts as an anti-apoptotic protein, decreased in several DLBCL cell lines after treatment with chidamide at concentrations up to 10 µM [51].

Rituximab, a monoclonal antibody therapy, has been shown to induce apoptosis in B-cell lymphoma. B-cell lymphoma sensitivity to rituximab-induced apoptosis can be augmented by hampering BCL-2 family proteins [55]. Inhibiting BCL-2 expression via STAT3 downregulation may be a potential target for chidamide therapy, suggesting a synergistic anticancer combination drug for B-NHL with high STAT3 and BCL2 expression. However, the basic finding described above requires further research into the use of chidamide in B-cell lymphoma with high STAT3 and BCL2 expression. The effect of apoptosis induced by immuno- and chemotherapeutic agents is expected to be enhanced after administration of chidamide. Therefore, further in vivo studies and clinical trials are required to confirm the promising results of previous in vitro studies.

Chidamide’s effect on apoptosis was confirmed in vivo, and the results were consistent with in vitro findings. Following chidamide administration, PI3K/PDK1/AKT axis was disrupted, resulting in decreased STAT3 activity and BCL-2 expression and increased apoptosis. A western blot revealed a significant reduction in PDK1 and phospho-AKT expression after chidamide treatment for 36 h, indicating chidamide’s inhibitory activity on the PI3K/AKT pathway. Cancer cell signaling pathways are complex, with the PI3K/AKT pathway and its downstream protein mTOR regulating cell growth and death in many types of cancer [59, 60]. Chidamide can partially induce apoptosis in transformed FL cells by interfering with the PI3K/AKT signaling pathway, thereby decreasing mTOR expression followed by attenuating of STAT3 and BCL-2 activities. Cell death occurs in a dose- and caspase-dependent manner. The final outcome of this mechanism was caspase-dependent apoptosis, as evidenced by the clear appearance of cleavedPARP and caspase 3 in the western blot in a time-dependent manner [40]. Based on the previous explanation about chidamide mechanism of action, BCL-2 is a promising target of chidamide which can be inhibited to limit tumor growth. The mechanisms which have been described are in line with the results of a clinical trial conducted by Fang et al. in DLBCL patients with double BCL2/MYC expression, in which this specific group of patients received more benefits after administration of chidamide and doxorubicin [58].

Given the presence of many cancer-related features, not all B-NHLs are susceptible to anticancer agent-induced apoptosis. Some of them are resistant to various signaling pathways, preventing apoptosis [59]. Several B-NHL subtypes may develop resistance to apoptosis when standard chemotherapy and immunotherapy are used. In this case, another mechanism of tumor cell death could be an alternative option. In the resistant Burkitt lymphoma model, autophagy developed following chidamide treatment via upregulation of B-cell translocation gene-1 (*BTG1*). Chidamide caused acetyl histone H3K9 to bind to the *BTG-1* promoter, suggesting that *BTG1* could be a potential chidamide target gene [43]. Chidamide has an interesting ability to induce cell death via autophagy, as evidenced by the low levels of the autophagy biomarker p62 after chidamide administration to lymphoma B-cells that are refractory to existing standard treatments. The mechanism underlying autophagy is the upregulation of *BTG1* expression, which is caused by acetylated histone H3K9 binding to the *BTG1* promoter [43]. According to this study, the role of chidamide as an epigenetic modifier by increasing histone acetylation in overcoming drug resistance is becoming clearer.

#### Immunological reprogramming of tumor microenvironment

Survival of cancer cells depends on the condition of the tumor microenvironment (TME), including immune cells, extracellular matrix, blood vessels, and stromal cells [60]. The TME is not simply a silent observer, but rather an active promoter of cancer progression [61]. Some costimulatory and coinhibitory molecules are present in the TME and interact with each other to support the development of tumor cells, such as programmed cell death protein 1 (PD1), programmed death-ligand 1 (PDL-1), and cytotoxic T-lymphocyte-associated protein 4 (CTLA-4). PD1 and PDL-1 can interact and allow the tumor cells to keep growing. To overcome this event, PD1 and PDL-1 antibodies can prevent their interaction and allow cytotoxic T cells to kill tumor cells. Chidamide together with PD1, PDL-1, and CTLA-4 antibodies, have been found to significantly inhibit tumor progression in an A20 mouse model compared with the administration of a single modality [62].

The TME condition changed after administration of chidamide and immune checkpoint inhibitor (ICI)–chidamide combination. Chidamide alone can increase the population of dendritic cells and natural killer cells. In addition, chidamide together with anti-PD-1 can increase the population of other immune cells, such as macrophages and cytotoxic T cells. Bissonnette et al. reported many genes involved in changing TME conditions. Chidamide can increase the work of ICI PD-1 by strengthening gene expression of PD-1, PDL-1, and CTLA-4, thereby significantly reducing tumor volume in in vivo models with both combinations of chidamide and ICI PD-1 [62].

Chidamide with PD-1 antibody augmented the immunosurveillance in TME [62]. Major histocompatibility complex (MHC) class I and II act as cell surface proteins that play a role in antigen presentation to CD4+ T cells to initiate a specific immune response. More than 50% of patients with DLBCL and classic Hodgkin’s lymphoma have loss of MHC class I as well as MHC class II expression. in patients with DLBCL and other types of mature B-cell lymphoma [63, 64]. The level of MHC class II expression is known to correlate directly with the number of tumor infiltrating T-cells in DLBCL so that it is also related to patient survival [65]. Because MHC class I and MHC class II expression are frequently decreased by epigenetic mechanisms in cancers, HDACIs, such as, trochostatin A, sodium butyrate apicidin, and entinostat, can increase the expression of MHC class I [66]. Chidamide and its combination with PD-1 antibody can also enhance several MHC I and MHC II classes in order to improve immunosurveillance [62]. Cycon et al. reported the mechanism of action of HDACIs in upregulating not only MHC class I but also MHC class II. MHC class II expression decreased in DLBCL cells due to the downregulation of MHC class II transactivator (CIITA) as the main regulator of MHC class II transcription. Histone modification using HDAC1 specific inhibitor can significantly increase CIITA and MHC II expression in DLBCL, thus preventing tumor progression [65].

The effector responses to kill the tumor cells and alteration of TME toward inflammation were enhanced by chidamide and the combination with PD-1 antibody [62]. The genes of *GZMB* which encodes granzyme B protein and *PRF1* which encodes perforin-1 increased significantly on days 7 and 14 after administration of the combination of chidamide and anti-PD-1 [62]. Granzyme B is a cytotoxic granule from adaptive and innate immune cells that directly cleaves and activates caspase resulting in apoptosis of tumor cells, while perforin creates pores at the cell membrane as the entrance of granzyme B [67]. Subsequently, other genes were involved to disrupt the TME after the combination treatment of chidamide and PD-1 antibody. Tumor-infiltrating lymphocyte recruitment, initial cytokine or CD8 effector response, and effector and memory cell differentiation occurred by modulation of several genes after chidamide treatment, including *41BB/CD137*, tumor necrosis factor alpha (*TNFA*), interleukin 2 receptor alpha (*IL2RA*)/*CD25*, *GZMB*, *IRF4*, chemokine (C-X3-X motif) receptor 1 (*CXC3R1*), chemokine (CXC motif) receptor 6 (*CXCR6*), and *CXCR3* [62]. In another study, *CXCR6*-deficient mice with solid tumor showed poorer control of tumor progression by CD8 T-cells [68]. CXCR3 functions as a receptor of CXCL10 ligand that provide chemoattraction of CD8+ and CD4+ effector T-cells to the tumor sites. It also induces the proliferation and function of T helper 1 effector. Moreover, this ligand can bind to the CXCR3 in the tumor cells and directly inhibit the tumor growth [69]. Therefore, we concluded that the modulation of *CXCR6*, *CXCR3*, and other genes by chidamide and ICIs was important for immunisurveillance that limited tumor progression and changed the TME from immunologically nonresponsive to responsive.

Chidamide could play a synergistic role in upregulating CD20 mRNA and protein expression levels by inhibiting HDAC3. When CD20 proteins are upregulated, rituximab, as a monoclonal CD20 antibody, may act more effectively to promote rituximab-mediated cytotoxicities, such as antibody-dependent cell cytotoxicity and complement-dependentcytotoxicity [14]. The mechanism of action of chidamide in killing the tumor cells via enhancing antibody-dependent cell cytotoxicity and complement-dependent cytotoxicity will be discussed further in the next section.

### Combination of chidamide with other treatments

#### Doxorubicin

Double-expressor lymphoma is defined as DLBCL that displays coexpression of MYC and BCL-2 proteins by immunohistochemistry. This B subtype has a poor prognosis after standard chemoimmunotherapy, high-dose chemotherapy with autologous transplantation, or with allogeneic transplantation [70]. MYC expression in lymphoma can promote genomic instability, gene amplification, and cellular proliferation. However, it can also repress apoptosis by increasing the expression of *TP53*, a tumor suppressor gene. MYC needs another genetic events including expression of BCL-2 and mutation in *TP53* to induce sustained proliferation and oncogenesis. Therefore BCL-2 overexpression is synergistic with MYC and another oncogene to promote the progression of lymphoma and chemotherapy resistance [71]. Fang et al. reported that chidamide in combination with doxorubicin can significantly promote growth inhibition in patient-derived xenograft models with DEL. Chidamide was revealed to have synergistic effect with doxorubicin to regulate a set of genes associated to multiple signaling pathways involved in the regulation of MYC and BCL-2. Those pathways are IL-6-JAK-STAT and PI3K-AKT-mTOR pathways. Moreover, using Assay for Transposase-Accessible Chromatin using sequencing, chidamide and doxorubicin were indicated to corepress the enhancers associated with lymphocyte development [58]. As previously described, both of these pathways clearly contributed to the progression of lymphoma, and chidamide can retard these pathways through several mechanisms (Figure 4).

#### Venetoclax

Chidamide, an HDACI, can reduce cMYC and *TP53* expression while increasing BIM expression, a proapoptotic protein. As previously stated, BCL-2 plays an important role in DLBCL apoptosis resistance. Venetoclax is an anticancer agent with a strong affinity for and inhibitory effect on BCL-2 in tumor cell mitochondria. Chidamide and venetoclax can synergistically promote apoptosis by increasing BIM protein expression and reducing BCL-2 family protein activity. However, human clinical trials are required to confirm these promising preclinical findings [44].

#### Cladribine, gemcitabine, and busulfan (CGB)

Cladribine is an antineoplastic purine analog that can incorporate into DNA, causing single-strand DNA breaks, decreasing nicotinamide adenine dinucleotide and adenosine triphosphate, and promoting apoptosis. It is selectively toxic to lymphocytes due to low deoxynucleotide deaminase activity, which can render antineoplastic drugs inactive [72]. Gemcitabine is a broad-spectrum antimetabolite and deoxycytidine analog with antitumor activity, which can terminate DNA replication in tumor cells [73]. Both disrupt DNA synthesis and repair, promoting apoptosis in B-cell lymphoma. Chidamide is essential for loosening and opening up DNA, making genomic DNA more susceptible to busulfan crosslinking. The combination of chidamide and CGB causes DNA damage via the ATM signaling pathway and increases the permeability of the mitochondrial membrane, allowing many proapoptotic proteins to leak into the cytoplasm. The final result of these pathways is a tumor cell apoptotic cascade [26].

#### Lenalidomide

Lenalidomide is an immunomodulatory agent that is frequently used to treat B-NHL. It can reverse the tumor-supporting network within the local microenvironment in DLBCL, FL, and MCL [74]. Many surface membrane proteins and costimulatory molecules are involved in establishing the TME. For example, low expression of CD20 and MHC II in tumor cells is a real obstacle to curing patients with R/R DLBCL that must be overcome [75]. Some HDACIs can restore MHC II expression, which is low in B-cell lymphoma. Lenalidomide and HDACIs have similar immunomodulatory effects and are thought to synergize in antitumor immunity by increasing the expression of MHC class I and II, as well as costimulatory molecules [76].

#### Rituximab

Approximately 26% of patients shifted from CD20 positive to CD20 negative after rituximab treatment, as detected by quantitative reverse transcription polymerase chain reaction, immunohistochemistry, and flow cytometry. These findings suggested that some epigenetic mechanisms have a role in decreasing CD20 expression [8]. HDAC3 and 6 play a role in the epigenetic mechanism that renders the *MS4A1* gene inactive, which encodes CD20 expression, [13, 77]. Chidamide, when used alone, can inhibit HDAC3 and thus increase CD20 expression at both the transcriptional and translational levels [14]. Although chidamide alone cannot inhibit HDAC6, it has been reported to inhibit HDAC6 more effectively when combined with venetoclax [78]. Taking this into account, CD20 can be increased using chidamide alone or in combination with other drugs. However, future studies are required to confirm whether venetoclax combined with chidamide can also amplify CD20 expression in B-cell lymphomas with CD20 negative conversion in vitro and in vivo.

Patients receiving rituximab monoclonal antibody therapy benefit from increased CD20 expression. After rituximab administration, lymphoma B-cells become more sensitive to complement-dependent cytotoxicity and antibody-dependent cell cytotoxicity by increasing CD20 expression [13, 79]. complement-dependent cytotoxicity can increase cell death by autolysis through the formation of a membrane attack complex, whereas antibody-dependent cell cytotoxicity can kill cancer cells via intermediary natural killer cells that secrete chemical cytokines that induce apoptosis in tumor cells [80]. Therefore, the effect of immunotherapy can be increased by modifying the epigenetic mechanism in lymphoma B-cells.

### Chimeric antigen receptor (CAR) T-cells

Patients with relapsed and refractory B-cell lymphoma still have a poor prognosis, with long-term survival rates of only 20%–40% [81]. Chimeric antigen receptor (CAR)-T-cell therapy is a novel treatment for B-NHL that significantly increases remission rates in relapsed and refractory patients. CAR-T-cell therapy uses genetically modified T cells to express chimeric antigen receptors, allowing them to kill cancer cells that express specific target antigens [82]. The target antigen expressed by B-NHL is CD19. However, further data revealed that the expression of CD19, the CAR-T-cell target, was lost after a series of CAR-T-cell treatments [83, 84]. This makes it difficult for hematologists to use CAR-T-cells as B-NHL therapy. Instead, CD22-targeting CAR-T-cells were further developed to overcome this problem. A research report showed that CAR-T-cells can restore remission rate in 50%–80% of patients who relapse due to loss of the CD19 target antigen after CAR-T-cell therapy [85]. In addition, CD22 was lost from the cell surface after a series of CD22 CAR-T-cell therapies [86].

Chidamide has been reported to increase CD22 expression in lymphoma B-cells. Chidamide, as an HDACI, can regulate post-transcriptional modifications that affect CD22 transport and redistribution, thereby increasing CD22 expression on the cell surface. Yang et al. concluded that chidamide and CD22 CAR-T-cell immunotherapy may enhance CAR-T-cell ability to cure B-NHL with reduced or lost CD22 target antigen [87].

### Chidamide application in clinical settings

The outcomes of patients with B-NHL who received a drug combination with chidamide were better than those of patients who received chidamide alone. The response of patients with R/R DLBCL who received single chidamide therapy was still low (overall response rate [ORR 25%]) compared to the combination regimen of chidamide and rituximab (ORR 40%) [23, 27]. This result might be due to the fact that lymphomagenesis, relapse, and refractory events are not only controlled by an epigenetic mechanism. Some hallmark capabilities of tumor cells, such as genome instability and mutation, deregulating cellular metabolism, avoiding immune destruction, and resisting cell death, also contribute to the development of malignancies [59]. Therefore, other drugs that target each hallmark of the tumor cells are also needed to obtain better outcomes in B-NHL patients.

Not all B-NHL patients are sensitive to chidamide. Some patients are resistant to chidamide and will not receive significant improvement with chidamide. Sun et al. reported that *CREBBP*-proficient DLBCL cells were more resistant to chidamide than *CREBBP*-deficient DLBCL cells. Because DLBCL patients with *CREBBP* deficiency showed a more favorable response to chidamide administration, we concluded that *CREBBP* loss of function that alters epigenetic activity can be used as a biomarker to predict sensitivity to chidamide treatment [27]. Jiang et al. [88] explained that *CREBBP* mutations induce deacetylation by BCL6/SMRT/HDAC3 complexes binding to enhancers of B-cell signaling and immun-response genes. Inhibition of HDAC 3 by chidamide can restore enhancer histone acetylation, allowing the tumor suppressor function of CREBBP in DLBCL to function well. Consistent with the results of a phase II clinical trial, Zhang et al. [24] reported that chidamide may mitigate the negative prognostic effect of *CREBBP/EP300*-mutant DLBCL. Therefore, we conclude that patients with *CREBBP*-mutant DLBCL can be well treated with an epigenetic drug that specifically inhibits HDAC3.

Chidamide was found to be more effective for DLBCL treatment when combined with R-CHOP. The response rate of patients with new DLBCL was slightly higher in the chidamide plus R-CHOP (CR-CHOP) group (ORR 94%) than in the R-CHOP alone group (ORR 90%). The 2-year overall survival (OS) was also higher in the CR-CHOP group (OS 83%) than in the R-CHOP group (OS 70%) [24, 89]. However, the baseline characteristics of the patients in these two studies were different. Therefore, an ongoing phase III clinical trial comparing two different groups is required [90].

The standard treatments for R/R aggressive B-cell lymphoma are high-dose therapy and autologous stem-cell transplantation (HD-ASCT) [91]. The salvage treatments that gave the best response were rituximab plus cisplatin, cytarabine, dexamethasone, etoposide, ifosfamide, methotrexate (R-DHAP-VIM) (ORR 75%; 2-year progression-free survival [PFS] 52%) and rituximab plus ifosfamide, carboplatin, etoposide (R-ICE) (ORR 53%; 2-year PFS 54%) after ASCT [92, 93] The chidamide regimen with CGB (Chi-CGB) outperformed the R-DHAP and R-ICE regimens in terms of complete response, PFS, and OS [26]. Consistent with another study that used dual epigenetic agents as a salvage treatment, chidamide and decitabine plus rituximab, gemcitabine, and oxaliplatin (CD-R-GemOx) provided a good disease control response in patients with R/R DLBL [28]. These findings suggest that the Chi-CGB and CD-R-GemOx regimens have promising effects as pre-transplant conditioning therapies in patients with aggressive R/R B cell lymphoma. However, a phase III clinical trial investigating these regimens in a larger population is warranted.

Not all patients can receive HD-ASCT because of age, comorbidities, and the patient’s unwillingness to receive HD-ASCT [94]. According to current guidelines, patients with severe comorbidities or psychiatric illness, active central nervous system, or HIV seropositivity are ineligible for ASCT. Other criteria for ineligibility for ASCT include an abnormal forced expiratory volume in the first second, bilirubin, creatinine level, or low cardiac ejection fraction [95]. The most promising treatment options came from phase I/II clinical trials with novel and experimental drugs [91]. A promising result of chidamide plus prednisone, etoposide, lenalidomide (PEL) use for patients with R/R DLBCL who are ineligible for intensive chemotherapy and ASCT has been reported. However, the optimal schedule to administer this drug combination has yet to be determined [25].

Based on the mechanism of action revealed in preclinical studies, chidamide has promising antitumor activity. Various chidamide doses (10, 20, 30, and 40 mg) were used in the trials. The patient’s age appears to be a factor in determining the dose. Most of these phase II studies concluded that chidamide combinations showed good results as first-line, second-line, or salvage therapy in patients with B-NHL [23–26, 28, 29]. Because all existing reports present data from single-arm studies with relatively small samples, further studies involving larger participants and control groups are needed to confirm these positive results.

The results of the phase II trials on chidamide utilization as advanced therapy for patients with R/R B-NHL show favorable outcomes with tolerable AE in a single or combination therapy. These findings are consistent with previous preclinical studies that demonstrated chidamide’s ability to inhibit B-NHL growth via a variety of mechanisms. The dose of chidamide that can be well tolerated is 30 mg twice a week. A grade III AE (neutropenia) occurred in 14.3% of patients, while no thrombocytopenia and minimal severe nonhematologic AE occurred; none of the patients experienced grade IV treatment-emergent AE after chidamide administration [29]. In contrast to chidamide, vorinostat, a first in class pan-HDACI approved for treating cutaneous T cell lymphoma showed grade ≥ III AEs in the form of thrombocytopenia (48%) and neutropenia (41%). Moreover, severe nonhematological AEs still occurred in 20% of 56 patients with B-NHL [96].

Chidamide in combination with other chemotherapy regimens also shows a promising safety profile vs another HDACI. The largest clinical trial examining HDACI vorinostat as combination therapy with the R-CHOP regimen showed grade III–IV hematological AEs as neutropenia (63%), febrile neutropenia (38%), thrombocytopenia, and anemia (36%) in the participants [97]. Additionally, chidamide and R-CHOP were associated with grade III–IV hematological AEs, including neutropenia (84%), febrile neutropenia (12%), thrombocytopenia (10%), and anemia (18%). However, the grade III–IV AEs experienced in participants who received the combination of chidamide and R-CHOP appeared to be better tolerated than the combination of vorinostat and R-CHOP, which was associated with death of the one patient during the study period [24, 97]. Other clinical trials also showed that administration of chiamide in combination with several other regimens, such as rituximab; CGB regimens; and decitabine, rituximab, gemcitabine, and oxaliplatin, could be well tolerated by patients with R/R DLBCL [23, 26, 28]. However, due to the potential for research bias in study design and the number of participants, the use of chidamide with prednisone, etoposide, and lenalidomide regimens needs to be investigated further in a prospective cohort study design involving a wider range of participants before drawing conclusions [25]. Epigenetic mechanisms, which have become one of cancer’s hallmarks, could be a potential target for combination with other anticancer and immunotherapy drugs. Taking this into account, patients receiving the chidamide-containing regimen are expected to have better clinical outcomes with fewer side effects in R/R B-NHL.

## Conclusion

Nonmutational epigenetic alterations have been identified as a hallmark of cancer, including B-NHL. Chidamide, as an HDACI, has the ability to limit growth, proliferation, and survival of the tumor cells, as well as promote apoptosis and autophagy in B cell lymphoma via various epigenetic and nonepigenetic modifications. Consistent with preclinical studies, chidamide has shown promising results in clinical trials over the last decade as a combination therapy with existing regimens in patients with new and relapse/refractory B-NHL. To recommend chidamide use in patients with B-NHL in daily practice, further clinical trials on a larger population with well-randomized designs are needed.
